# Characteristics of undergraduate and second speciality mental health programmes in Peru: a cross-sectional study

**DOI:** 10.1186/s12960-023-00805-9

**Published:** 2023-03-01

**Authors:** Jackeline García-Serna, Guillermo Almeida-Huanca, Jeff Huarcaya-Victoria, Ana Lucia Vilela-Estrada, Jessica Hanae Zafra-Tanaka, David Villarreal-Zegarra

**Affiliations:** 1 Instituto Peruano de Orientación Psicológica, Lima, Peru; 2 Escuela de Medicina, Universidad Científica del Sur, Lima, Peru; 3 Escuela Profesional de Medicina Humana, Universidad Privada San Juan Bautista, Filial Ica, Ica, Peru; 4 Unidad de Psiquiatría de Enlace, Departamento de Psiquiatría, Hospital Nacional Guillermo Almenara Irigoyen, EsSalud, Lima, Peru; 5 South American Center for Education and Research in Public Health, Universidad Privada Norbert Wiener, Lima, Peru

**Keywords:** Education Medical, Education Nursing, Health personnel, Community Mental Health Centers

## Abstract

**Background:**

This study aims to describe the training offered and the availability of professionals required by the Ministry of Health for mental health problems management in the community.

**Methods:**

A cross-sectional study was carried out on the training offered in mental health in Peruvian universities. A search for programs was conducted using the University Information System database and universities' websites, as well as using the Ministry of Health's database on health personnel and data on the number of enrolled and current students provided by the University Information System database and the Transparency section of the universities.

**Results:**

There were 214 undergraduate, 55 specialty and 7 subspecialty programmes, of which 39%, 47% and 100%, respectively, were offered in the capital city. The duration ranged from 5 to 7 years for undergraduate programs and from 1 to 3 years for subspecialty and second specialty programs. The cost of undergraduate programs ranged from free of charge up to USD 6863.75 for the first semester of study. Second specialty programs ranged from 720 up to 11 986 USD and subspecialty programs ranged from 2267 up to 9138 USD, with medicine being the most expensive. On the other hand, there are a greater number of psychology students (*n* = 78 781) pursuing undergraduate studies than working professionals (*n* = 5368), while in the second specialty of psychiatry there are far fewer students pursuing the specialty (*n* = 67) than working professionals (*n* = 454).

**Conclusions:**

The problem of professional training in mental health requires that the institutions involved in health and education develop policies to decentralize programs, communicate the demand for professionals in certain areas, make them accessible to the low-income population, respond to mental health problems and guarantee their quality. On the other hand, regarding the low number of mental health personnel working, it is suggested to increase the mental health budget to generate more mental health services and employment.

**Supplementary Information:**

The online version contains supplementary material available at 10.1186/s12960-023-00805-9.

## Background

Globally, there are 9 mental health professionals (psychiatrists, nurses, psychologists, social workers, occupational therapists or speech therapists) per 100 000 inhabitants [1]. Whereas in South America, there are 8.8 mental health professionals per 100 000 inhabitants, with a higher number of psychologists (8.6/100 000) as opposed to psychiatrists (2.4/100 000) [2]. On the other hand, in Peru, with 31 million inhabitants in 2017 [3], there were 2.95 psychiatrists, 0.21 child psychiatrists, 1.02 mental health nurses, 9.51 psychologists, 0.36 social workers, 0.09 occupational therapists and 0.12 speech therapists per 100 000 inhabitants [4]. In this context, it was reported in the literature that South America has a treatment gap of 73.1% [5]. Likewise, the lack of attention to LMICs could affect the individual, social and economic well-being of the child population [6].

The Peruvian Ministry of Health planned and implemented the National Human Resources Policy [7] with the objective of (a) "Having sufficient Human Resources in Health, reducing inequity" and (b) "Strengthening the professional competencies of human resources that respond to the needs, demands and expectations in health" [8]. The first objective was achieved progressively, as the number of human resources increased, although it is not equitable at the geographical and socio-economic levels. To achieve the second objective, the Ministry of Health and the Ministry of Education developed normative activities, but this was insufficient and hindered the planning of the quantity and quality of personnel required for the health system. Likewise, the Ministry of Health did not complete the formulation of job competency profiles required as a minimum for a basic health team, limiting the orientation of training [8].

In Peru, mental health professionals have different requirements to practice their profession. Psychologists and medical technologists must obtain a professional degree, which consists of completing their undergraduate studies and submitting a thesis, as well as being registered with their respective professional associations [9, 10]. However, job opportunities may require specialisation studies in some area of mental health as a second specialisation [11] which is of lower rank than a master's degree. In contrast, doctors and nurses require a second specialisation in mental health in addition to a bachelor's degree [11, 12].

For countries to ensure sufficient availability of personnel, they must have a certain number of professionals applying and graduating [13]. Likewise, given the need for mental health professionals, it is necessary to have a sufficient number of educational programmes [14] that respond to the needs of institutions that provide mental health care. One of the institutions that provide specialised care in Peru is the Community Mental Health Centres which are distributed nationwide and were the result of the mental health reform [15].

These centres offer specialised care for mental health problems. They have specialised services for different age groups (from children to the elderly), specialisation in addictions and social and community participation. Health care is based on a territorial approach for approximately 100 000 inhabitants. The personnel required are psychiatrists, family doctors, psychologists, nurses, social workers and medical technologists (specialised in speech therapy and occupational therapy). In addition, the services they offer require psychologists and nurses to have certain specialisations [11].

In addition to the need to be aware of the number of programmes that exist to ensure the availability of health professionals, other characteristics may make them less accessible. One of those is geographic location; since education is seen as a way out of poverty, many young people migrate in pursuit of better education [16]. However, it is not certain that these professionals will return to their place of origin, as cities may offer more employment opportunities [17]. The type of management of the universities offering the programmes is also relevant, since although public universities offer free education, they are very selective due to their demand [18]. While private universities, which are not always rigorous in the selection of applicants, have costs of over 111.95 dollars per month, which varies according to the programme and university [19].

It has also been reported that the economic aspect, which involves the cost of the programmes, is a triggering factor for the interruption of studies, where 47.7% of Peruvian students who interrupted their studies in 2018 were from the lowest socio-economic level. It is important to have sufficient financial resources to study 120 credits, equivalent to 3 years if studying a technical degree [20], and 5 years if studying an undergraduate university degree [21] in Peru. It is also possible that certain careers have more training opportunities and are more accessible than others.

In Peru, mental health problems continue to be one of the main causes of loss of healthy life years due to the disability they generate [22]. Furthermore, 210 Community Mental Health Centres nationwide require mental health staff [23]. It is, therefore, important to investigate the availability of mental health staff and the supply of training to renew it. This study aims to describe the training offered and the availability of professionals required by the Ministry of Health for the management of mental health problems in the community [11].

## Methods

### Design

A cross-sectional study was conducted using secondary data sources. Data were collected from undergraduate and second specialty (specialty and subspecialty) mental health programmes in Peruvian universities at the national level and then contrasted with the number of staff available in Ministry of Health facilities. The second speciality is a type of training focused on the development of clinical skills with a minimum duration of two semesters and requiring the approval of a thesis or academic work [21]. In addition, medical residencies are equivalent to a second speciality, which can be either specialties or subspecialties [24], where the first lasts longer and is a prerequisite for a subspecialty.

### Eligibility criteria

The selected programmes were offered by Peruvian universities authorised by the Superintendencia Nacional de Educación Superior Universitaria (SUNEDU), which authorises universities based on quality standards [25]. We selected programmes that meet the mental health staffing needs required by Community Mental Health Centres [11].

Thus, programs in medicine, nursing, psychology, social work, and medical technology (with emphasis on occupational therapy and speech therapy) were evaluated. For the second specialties, our choices were psychiatry, addiction psychiatry, child and adolescent psychiatry, and community family medicine (targeted to physicians); the second specialization in mental health and public health (targeted to nurses); the second specialization in cognitive–behavioral therapy and family therapy (targeted to psychologists); and speech therapy (targeted to general health personnel).

### Sources of data

The University Information System database https://www.tuni.pe/ (created by SUNEDU) was used to identify the licensed universities and the programmes they offered. The results of the identified programmes were cross-checked with the search through each university's website. The data extracted were (1) name of the university, (2) name of the programme, (3) region of origin, (4) target population, (5) duration in years, (6) university management (public or private), (7) minimum cost/study cost (monthly fees and tuition) and (8) maximum cost/total cost (tuition, monthly fees and additional costs). The minimum and maximum cost refers to the cost of undergraduate programmes, as the same university may offer a range of prices for the same programme. While the cost of study and total cost refers to second specialisations, where additional costs refer to the cost of applying for a second major or other.

To make the comparison with the number of available professionals, the database on current health personnel provided by the Observatory of Human Resources in Health for the years 2021 and 2022 was used [26]. The information on the number of students entering and enrolled in training programs was obtained from two sources, both from the University Information System [27] in its tab called databases and from the Transparency portal located on the website of each university.

### Procedure and data analysis

First, authorized universities were searched on the University Information System platform and 94 universities were found. This number did not change until the final date of data collection (March 2021 to October 5, 2021). The same platform was then searched for programmes that met the eligibility criteria, these results were cross-checked by searching the websites of each university (Additional file 3) and, finally, the data were extracted. In addition, databases on available healthcare staff and the number of incoming and enrolled students were downloaded and adapted for further analysis. The descriptive analysis was carried out using Excel and R [28]. Tables are presented with a summary of the characteristics of the training programmes by level and a comparison of the number of existing programmes, the number of professionals enrolled and the available staff of the Ministry of Health. Regarding the price, its equivalent was generated with the Peruvian minimum living wage (SMV), which is USD 238.68 (exchange rate of 12 January 2022) [29].

### Ethical issues

Our protocol was approved by the Institutional Research Ethics Committee of the Instituto Peruano de Orientación Psicológica (Peruvian Institute of Psychological Orientation). Our study does not collect primary data from individuals but collects open data on training programmes in Peruvian universities. Therefore, there is no corresponding ethical risk for participants or institutions. Furthermore, our study is aligned with the norms of the Declaration of Helsinki.

## Results

### Undergraduate mental health training programmes

**Fig. 1:**
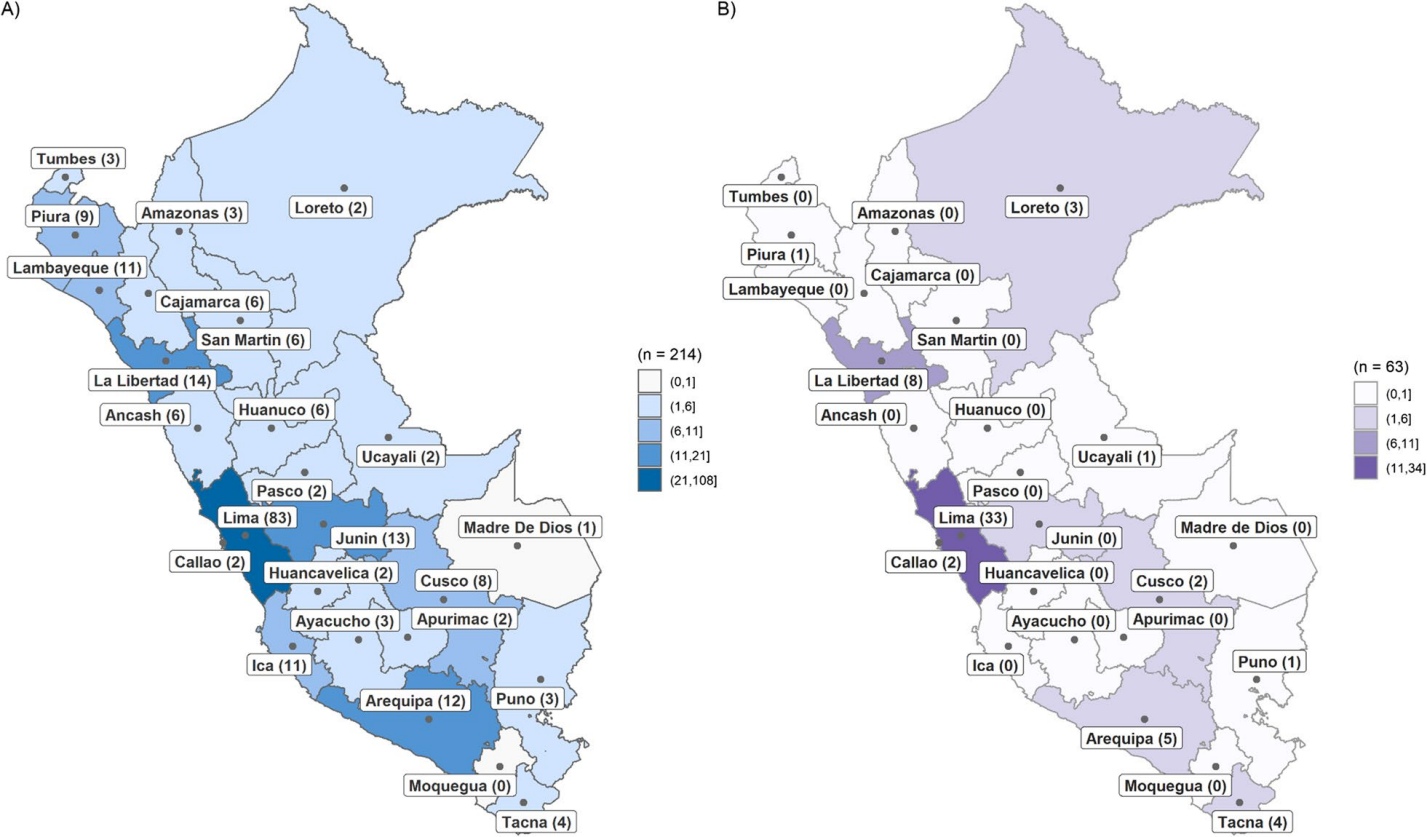
Distribution of undergraduate and second speciality programmes by region. **A** Undergraduate programmes by region. **B** Second speciality programmes by region

At the national level, 214 undergraduate programmes were found to be offered in 64 universities (Fig. 1). The characteristics of each of these programmes are listed in Table 1. Thirty-nine per cent of the national offerings in mental health education are located in the country's capital, mostly medical technology and psychology programmes, while 33% of all undergraduate programmes in mental health are offered by public universities, mostly social work programmes. Regarding costs per semester, private universities can range from 1.8 to 29.5 minimum wages (USD 409.50 to USD 6863.75), depending on the degree programme, with medicine and psychology programmes being more expensive. On the other hand, the duration of the programmes ranges from 5 to 7 years. Additional file 1 shows the specific characteristics of each degree program (costs, duration, and institution offering the program).

**Table 1:** Summary of programme characteristics

	Medicine	Psychology	Nursing	Social work	Medical technology	Multidisciplinary	Total
Undergraduate							
Number	48	81	70	11	4	–	214
% Program in Capital city	35%	46%	31%	27%	100%	–	39%
% Public program	42%	10%	46%	82%	50%	–	33%
Range of semestral cost in the public program in USD (MLW)	0–25 (0–0.1)	0–25 (0–0.1)	0–25 (0–0.1)	0–12.50 (0–0.1)	0	–	0–25 (0–0.1)
Range of semestral cost in the private program in USD (MLW)	753.75–6863.75 (3.2–29.5)	450.50–5827.50 (1.9–25.1)	409.50–1723.75 (1.8–7.4)	499–662.50 (2.1–2.8)	1326.25–1678.75 (5.7–7.2)	–	409.50–6863.75 (1.8–29.5)
Range of time	7	5–6	5	5	5	–	5–7
Second speciality							
Number	31	4	16	–	–	4	55
% Program in Capital city	45%	50%	50%	–	–	50%	47%
% Public program	42%	50%	75%	–	–	25%	51%
Range of total cost in public programs in USD (MLW)	3233–9570.50 (13.9–41.2)	2637.50 (11.3)	720–2140 (3.1–9.2)	–	–	1737 (7.5)	720–9570.50 (3.1–41.2)
Range of total cost in Private program in USD (MLW)	7355.50–11 986 (31.6–51.6)	825 (3.5)	1183.75–3487.50 (5.1–15)	–	–	825–867.50 (3.5–10.5)	825–11 986 (3.5–51.6)
Range of time	3	1–2	1–2	–	–	1–2	1–3
Subspecialty							
Number	7	–	–	–	–	–	7
% Program in Capital city	100%	–	–	–	–	–	100%
% Public program	43%	–	–	–	–	–	43%
Range of total cost in public programs in USD (MLW)	2268–3550.50 (9.8–15.3)	–	–	–	–	–	2268–3550.50 (9.8–15.3)
Range of total cost in Private program in USD (MLW)	5238–9138 (22.5–39.3)	–	–	–	–	–	5238–9138 (22.5–39.3)
Range of time	2	–	–	–	–	–	2

MLW: Minimum living wage in Peru. 1 MLW = 930 PEN (USD 232.5). PEN: Peruvian Soles

^*^Medical Technology: focus on speech therapy and occupational therapy

### Second speciality and subspecialty training programmes in mental health

58 s specialty programs were found in the University Information System database, but three were eliminated, because they were not available on the websites of their respective institutions. Our analysis included 55-s speciality programmes, of which 47% are offered in the capital of Peru and 51% come from public universities, where doctors and nurses were the main recipients of the programmes. Regarding costs per semester, private universities can range from 3.5 to 51.6 minimum wages (USD 825 to USD 11 986), with medicine and nursing programmes being more expensive. On the other hand, the duration of the programmes ranges from 1 to 3 years. As for the subspecialty programmes, 7 programmes were found to be entirely for physicians, all located in the capital and with 43% coming from public universities, while the cost in private universities can range from 22.5 to 39.3 (USD 5238 to USD 9138) minimum salaries with a duration of 2 years. Additional file 2 shows the specific characteristics of each second speciality and subspecialty programme.

### Comparison between existing programmes, professionals in training and existing staff in the Ministry of Health

At the undergraduate level, the largest number of programmes is directed towards the psychology degree (*n* = 81), and in 2021 this degree obtained a higher flow of entrants (*n* = 32 171) and enrolled students (*n* = 78 781) compared to the number of entrants (*n* = 14 325) and enrolled students (*n* = 46 896) for the medical degree. While in the health system in the same year, there is a higher number of nurses followed by physicians (*n* = 40 757) and psychologists (*n* = 5368), which remains similar in 2022.

At the subspecialty level, the highest number of programmes is directed to the second speciality of community family medicine (*n* = 20), the second speciality and the speciality of mental health nursing had the same and higher flow (*n* = 123) of students studying these specialities. While in the health system during the same year, there is a greater number of nurses specialising in public health (*n* = 483), followed by psychiatrists (*n* = 454), and it remains similar for 2022. The comparison between training programmes, trainees (entrants, enrolled and ongoing trainees) and available staff in the health system is shown in Table 2. In addition, some second speciality programmes had missing data or provided data on the number of students who completed the speciality during 2019 and 2020, as these were not updated.

**Table 2:** Summary of training programmes, trainees and available staff in the health system

	Number of programmes	Program in Capital city (%)	Public program (%)	Entrants 2021	Student enrolled (First semester 2021)	Student enrolled (Second semester 2021)	Mental health staff available (2021)	Mental health staff available (2022)
Undergraduate Programmes								
Medicine	48	35	42	14 325	46 896	46 381	40 757	29 318
Psychology	81	46	10	32 171	78 781	81 028	5368	5302
Nursing	70	31	46	16 308	32 968	34 590	47 509	46 961
Social work	11	27	82	792	3658	3440	2090	2090
Medical Technology*	4	100	50	37	189	202	316	415
Second Specialty program								
Psychiatry	11	55	36	–	67	52	454	386
Community Family Medicine	20	40	45	–	123	43	418	362
Child and adolescent psychiatry	5	100	40	–	3	5	12	13
Second speciality in addiction psychiatry	2	100	50	–	0	0	3	3
Second speciality in mental health nursing	8	50	75	–	123	155	264	278
Second speciality in public health	8	50	75	–	100	94	483	494
Systematic psychotherapy or other	3	33	33	–	21	1	39	51
Cognitive Behavioural Psychotherapy	1	100	100	–	95	0	32	45
Speech therapy	4	50	0	–	53	70	15	16

*Medical Technology: focus on speech therapy and occupational therapy

## Discussion

### Programme estimation and distribution

In Peru, there is an uneven supply and geographical distribution of training programmes at both undergraduate and second speciality levels. There are few undergraduate medical technology programmes in the area of occupational and speech therapy, as well as in social work, which is already reflected in the shortage of such personnel in Community Mental Health Centres [30]. This implies a danger for services dedicated to children and the elderly, so the Ministry of Health needs to make the demand for certain areas of medical technology visible [31]. On the other hand, the unequal distribution and concentration of programmes in the capital city reinforce migration for those who want to study these programmes and discourage those who want to remain in their regions of origin.

### Characteristics of undergraduate and postgraduate programmes

On the other hand, training programmes come mostly from private universities with costs above 409.50 USD, which can mean a barrier for low-income people. Although there is the possibility of receiving free education in public universities, admission is difficult due to high demand; a representative case is the Universidad Nacional Mayor de San Marcos (UNMSM), which in 2021 had 6891 vacancies, 49 386 applicants and 5977 undergraduate entrants [32]. Furthermore, the health measures established during the beginning of the pandemic reflected the economic fragility of the students, since during the first semester of 2020 the rate of interruption of studies was 18.3% and was reduced to 16.2% in the second semester of the same year [33].

Regarding the cost of programmes at private universities, this ranges from 1.8 to 51.6 salaries, which influences the choice of a training programme. Thus, for a group of medical students, the choice of a second speciality was mainly based on salary (23.6%), job opportunities (19.7%), and for a few on vocation (8.9%) [34]. In addition, access to universities with educational quality may be restricted to a certain sector of the Peruvian population. Currently, educational quality is often measured by rankings, including the QS World University Rankings [35], which in the 2022 edition included eight Peruvian universities, of which five (PUCP, UPCH, UNMSM, UPC, USIL) had health careers and only UNMSM was public [36].

The duration of the selected undergraduate training programmes varies from 5 to 7 years; 5 years being the minimum duration to obtain a bachelor's degree in Peru according to the University Law [21]. Therefore, if the labour market is not favourable, it is likely that more years of study will be required to increase skills; therefore, to favour the transition from education to work, the education system must guarantee the skills required to work [37].

Thus, there is also a need for teaching standards, for instance, the UK developed speciality programmes with criteria, such as the definition of the target population, the inclusion of evidence-based approaches and the development of core competencies of the chosen approach [38]. It is important that training is designed to prepare health care workers to perform their work in real-life conditions to avoid ineffective and inadequate outcomes during treatment and to benefit the recovery of users [39, 40].

On the other hand, although there are training programmes to generate mental health personnel to cover the population's need for care, there is also an economic barrier. Although the budget for mental health has been increasing since 2018, in 2022 it only represented 0.19% of the national budget and 1.6% of the budget allocated to the health function [41], which limits the generation of health services close to the community and jobs for mental health professionals [41].

### Implications for public health and education

Staff shortages impede the expansion of health care, the implementation of policies and the structuring of health systems [42]. One proposal to increase access to training programmes is to decentralise provision and assess the relevance of selection and admission criteria [43]. A benchmark for increasing access to higher education was Brazil, which financed a network of public universities and tried to consolidate distance education [44]. Another strategy to address geographical barriers is the e-learning model. However, this option requires access to the Internet, electronic devices [45], specialised staff to design the programmes, adapt e-learning to the reality of the participants [46] and train teachers [47]. In addition, to ensure the permanence of university students, scholarships can be implemented to help cover certain expenses of university life as Chile did [48] or to expand education by having the central or regional health system establish partnerships or support applicants with the payment of existing programmes in private universities.

On the other hand, task sharing is an alternative response to staff shortages that has been implemented in LMICs, expanding access to mental health care and decreasing the treatment gap [49]. Its approach is community-based and allows for collaboration between health professionals and non-specialists increasing the availability of staff. However, its effectiveness depends on funding, ongoing training and the active participation of community leaders and other stakeholders [50].

### Strengths and limitations

The strengths of this study are the search for programmes at the level of the campuses and branches of the universities, as well as the parallel search for programmes on the platform of the University Information System and the websites of the universities. In addition to this, it is possible to compare the information with the number of existing staff in the health system and the number of staff in training.

The first allowed us to estimate more accurately the number of programmes and the second allowed us to avoid overlooking newly created programmes. The third allows us to approximate the number of personnel required permanently compared to the number of personnel in training.

Regarding the weaknesses of the study, first, universities that were not licensed or were under evaluation by the National Superintendence of Higher Education were not considered. However, this should not affect the research considerably, as unlicensed universities usually cease their activities and their programmes cease to exist, while universities in the process of licensing have not yet demonstrated that they meet the minimum quality criteria. Second, the websites of some universities do not provide complete information on the programmes, but information on most of them could be found.

### Conclusions and recommendations

In Peru, there are few undergraduate programmes oriented towards social work and medical technology (focused on occupational therapy and speech therapy), as well as a few second speciality programmes for psychologists that respond to the needs of the community mental health centres. The first affects the possibility of having more professionals in health centres, while the second complicates the possibility of having professionals trained to work in specialised areas of mental health. Many programmes are centralised in the capital and offered in private universities whose programmes are equivalent to at least 1.8 minimum living wages per semester. While second speciality programmes are equivalent in total to at least 3.1 times the minimum living wage. We believe that to increase the number and quantity of competencies of mental health professionals, institutions involved in health and education should develop policies to decentralise existing undergraduate and second speciality programmes, make them more accessible to low-income people, and make them responsive to the needs of the population and guarantee the quality of all programmes involved in mental health.

The authors make two recommendations. First, to guarantee the quality of undergraduate and graduate mental health programs, it is necessary to strengthen the public institutions responsible for ensuring the quality of these programs, such as SUNEDU or the Peruvian Ministry of Education. Currently, there is an accreditation process for medical careers, but there are no guidelines for accrediting graduate or other undergraduate programs. Therefore, providing greater financial resources and independence to SUNEDU will allow it to extend guidelines for accrediting undergraduate and graduate programs. Second, to ensure that undergraduate and graduate programs in mental health are more accessible to low-income people, it is recommended that the National Scholarship and Educational Credit Program (PRONABEC) be strengthened, so that it can offer a greater number of scholarships to people with lower incomes. On the other hand, it is recommended to increase the offer of mental health programs in cities that do not have second specialty or subspecialty programs, such as Tumbes, Amazonas, Cajamarca, or Apurimac. Opening new programs in these cities could be an opportunity for licensed universities to conquer new markets.

## Supplementary Information

**Additional file 1.** Characteristics of undergraduate programs in dollars.

**Additional file 2.** Characteristics of second speciality programmes in dollars.

**Additional file 3.** Links to the websites of the universities and programmes collected.

## Data Availability

The data sets used and/or analysed during the current study are available from the corresponding author upon reasonable request.

## Abbreviations

MLW: Minimum Living Wage
PUCP: Pontificia Universidad Católica del Perú
UPCH: Universidad Peruana Cayetano Heredia
UNMSM: Universidad Nacional Nacional de San Marcos
UPC: Universidad Peruana de Ciencias Aplicadas
USIL: Universidad San Ignacio de Loyola
