# Supplementary material for: Characteristics of undergraduate and second speciality mental health programmes in Peru: a cross-sectional study
Source: Hum Resour Health. 2023 Mar 1;21:16. doi: 10.1186/s12960-023-00805-9 (PMC9979569; doi:10.1186/s12960-023-00805-9)
Supplement: Supplementary file 1 — Additional file 1. Characteristics of undergraduate programs in dollars. [file 12960_2023_805_MOESM1_ESM.docx]

Additional file 1. Characteristics of undergraduate programs in dollars.

| **MEDICINE** | | | | | | |
| --- | --- | --- | --- | --- | --- | --- |
| University | Location | Campus | Duration/ years (y), months(m) | Management type | Minimum Cost by semester (USD) | Maximum Cost by semester (USD) |
| Universidad Andina del Cusco | Cusco | central | 7 | private | 2,210.75 | 0.00 |
| Universidad Católica de Santa María | Arequipa | central | 7 | private | 1,567.00 | 2,089.00 |
| Universidad Católica Santo Toribio de Mogrovejo | Lambayeque | central | 7 | private | 2,356.25 | 0.00 |
| Universidad César Vallejo | La Libertad | central | 7 | private | 1,587.50 | 1,837.50 |
| Universidad César Vallejo | Piura | filial | 7 | private | 1,587.50 | 1,712.50 |
| Universidad Científica del Sur | Lima | central | 7 | private | 4,107.50 | 5,827.50 |
| Universidad Científica del Sur | Lima | filial | 7 | private | 4,107.50 | 5,827.50 |
| Universidad Científica del Sur | Lima | filial | 7 | private | 4,107.50 | 5,827.50 |
| Universidad Continental | Junín | central | 7 | private | 2,262.50 | 2,675.00 |
| Universidad Continental | Lima | filial | 7 | private | 2,825.00 | 3,100.00 |
| Universidad de Piura | Lima | filial | 7 | private | 3,767.50 | 5,312.50 |
| Universidad Nacional Daniel Alcides Carrión | Pasco | central | 7 | public | 0.00 | 0.00 |
| Universidad Nacional de Cajamarca | Cajamarca | central | 7 | public | 0.00 | 0.00 |
| Universidad Nacional de la Amazonía Peruana | Loreto | central | 7 | public | 0.00 | 0.00 |
| Universidad Nacional de Piura | Piura | central | 7 | public | 12.50 | 0.00 |
| Universidad Nacional de San Agustín | Arequipa | central | 7 | public | 0.00 | 0.00 |
| Universidad Nacional de San Antonio Abad del Cusco | Cusco | central | 7 | public | 0.00 | 0.00 |
| Universidad Nacional de San Cristóbal de Huamanga | Ayacucho | central | 7 | public | 0.00 | 0.00 |
| Universidad Nacional de San Martín | San Martin | central | 7 | public | 0.00 | 0.00 |
| Universidad Nacional de Trujillo | La Libertad | central | 7 | public | 12.50 | 0.00 |
| Universidad Nacional de Tumbes | Tumbes | central | 7 | public | 0.00 | 0.0022.2 |
| Universidad Nacional de Ucayali | Ucayali | central | 7 | public | 0.00 | 0.00 |
| Universidad Nacional del Altiplano | Puno | central | 7 | public | 0.00 | 0.00 |
| Universidad Nacional del Centro del Perú | Junín | central | 7 | public | 2.50 | 0.00 |
| Universidad Nacional del Santa | Ancash | filial | 7 | public | 22.25 | 0.00 |
| Universidad Nacional Federico Villarreal | Lima | central | 7 | public | 0.00 | 0.00 |
| Universidad Nacional Hermilio Valdizán | Huánuco | central | 7 | public | 15.00 | 25.00 |
| Universidad Nacional Jorge Basadre Grohmann | Tacna | central | 7 | public | 0.00 | 0.00 |
| Universidad Nacional José Faustino Sánchez Carrión | Lima | central | 7 | public | 0.00 | 0.00 |
| Universidad Nacional Mayor de San Marcos | Lima | central | 7 | public | 0.00 | 0.00 |
| Universidad Nacional Toribio Rodríguez de Mendoza de Amazonas | Amazonas | central | 7 | public | 15.00 | 0.00 |
| Universidad Peruana Cayetano Heredia | Lima | central | 7 | private | 4,116.25 | 5,803.75 |
| Universidad Peruana de Ciencias Aplicadas | Lima | central | 7 | private | 4,802.50 | 6,863.75 |
| Universidad Peruana Los Andes | Junín | central | 7 | private | 2,489.50 | 0.00 |
| Universidad Peruana Unión | Lima | central | 7 | private | 753.75 | 3,068.75 |
| Universidad Privada Antenor Orrego | La Libertad | central | 7 | private | 2,075.00 | 0.00 |
| Universidad Privada Antenor Orrego | Piura | filial | 7 | private | 2,075.00 | 0.00 |
| Universidad Privada de Tacna | Tacna | central | 7 | private | 1,536.25 | 0.00 |
| Universidad Privada Norbert Wiener | Lima | central | 7 | private | 2,337.50 | 0.00 |
| Universidad Privada San Juan Bautista | Lima | central | 7 | private | 3,020.00 | 3,270.00 |
| Universidad Privada San Juan Bautista | Lima | central | 7 | private | 3,020.00 | 3,270.00 |
| Universidad Privada San Juan Bautista | Ica | filial | 7 | private | 2,095.00 | 2,220.00 |
| Universidad Privada San Juan Bautista | Ica | filial | 7 | private | 2,095.00 | 2,220.00 |
| Universidad Ricardo Palma | Lima | central | 7 | private | 3,325.00 | 0.00 |
| Universidad San Ignacio de Loyola | Lima | central | 7 | private | 4,011.25 | 4,867.50 |
| Universidad San Martin de Porres | Lambayeque | filial | 7 | private | 1,689.90 | 1,987.55 |
| Universidad San Martin de Porres | Lima | central | 7 | private | 3,147.85 | 3,803.85 |
| Universidad Señor de Sipán | Lambayeque | central | 7 | private | 1,762.50 | 0.00 |

| **NURSING** | | | | | | |
| --- | --- | --- | --- | --- | --- | --- |
| University | Location | Campus | Duration/ years (y), months(m) | Management type | Minimum Cost by semester (USD) | Maximum Cost by semester (USD) |
| Universidad Andina del Cusco | Cusco | central | 5 | private | 755.75 | 0.00 |
| Universidad Autónoma de Ica | Ica | central | 5 | private | 557.50 | 782.503. |
| Universidad Autónoma de Ica | Ica | filial | 5 | private | 557.50 | 782.50 |
| Universidad Católica de Santa María | Arequipa | central | 5 | private | 480.00 | 960.00 |
| Universidad Católica Santo Toribio de Mogrovejo | Lambayeque | central | 5 | private | 953.75 | 1,402.25 |
| Universidad Católica Sedes Sapientiae | Lima | filial | 5 | private | 1,022.50 | 912.50 |
| Universidad Católica Sedes Sapientiae | Lima | central | 5 | private | 1,022.50 | 912.50 |
| Universidad Católica Sedes Sapientiae | San Martin | filial | 5 | private | 541.25 | 601.75 |
| Universidad César Vallejo | La Libertad | central | 5 | private | 587.50 | 837.50 |
| Universidad César Vallejo | Lima | filial | 5 | private | 650.00 | 775.00 |
| Universidad César Vallejo | Lima | filial | 5 | private | 625.00 | 750.00 |
| Universidad César Vallejo | Piura | filial | 5 | private | 587.50 | 712.50 |
| Universidad Científica del Sur | Lima | central | 5 | private | 1,263.75 | 1,626.25 |
| Universidad Científica del Sur | Lima | filial | 5 | private | 1,263.75 | 1,626.25 |
| Universidad Científica del Sur | Lima | filial | 5 | private | 1,263.75 | 1,626.25 |
| Universidad Continental | Junín | central | 5 | private | 722.50 | 928.75 |
| Universidad de Ciencias y Humanidades | Lima | central | 5 | private | 957.50 | 0.00 |
| Universidad de Huánuco | Huánuco | central | 5 | private | 443.03 | 0.00 |
| Universidad de Huánuco | Huánuco | filial | 5 | private | 409.61 | 0.00 |
| Universidad María Auxiliadora | Lima | central | 5 | private | 800.00 | 0.00 |
| Universidad Nacional Amazónica de Madre de Dios | Madre de Dios | central | 5 | public | 0.00 | 0.00 |
| Universidad Nacional Autónoma Altoandina de Tarma | Junín | central | 5 | public | 0.00 | 0.00 |
| Universidad Nacional Autónoma de Chota | Cajamarca | central | 5 | public | 0.00 | 0.00 |
| Universidad Nacional Autónoma de Tayacaja “Daniel Hernández Morillo” | Huancavelica | central | 5 | public | 0.00 | 0.00 |
| Universidad Nacional Daniel Alcides Carrión | Pasco | central | 5 | public | 0.00 | 0.00 |
| Universidad Nacional de Barranca | Lima | central | 5 | public | 0.00 | 0.00 |
| Universidad Nacional de Cajamarca | Cajamarca | central | 5 | public | 0.00 | 0.00 |
| Universidad Nacional de Cajamarca | Cajamarca | filial | 5 | public | 0.00 | 0.00 |
| Universidad Nacional de Cajamarca | Cajamarca | filial | 5 | public | 0.00 | 0.00 |
| Universidad Nacional de Huancavelica | Huancavelica | central | 5 | public | 8.75 | 0.00 |
| Universidad Nacional de la Amazonía Peruana | Loreto | central | 5 | public | 0.00 | 0.00 |
| Universidad Nacional de Piura | Piura | central | 5 | public | 12.50 | 0.00 |
| Universidad Nacional de San Agustín | Arequipa | central | 5 | public | 0.00 | 0.00 |
| Universidad Nacional de San Antonio Abad del Cusco | Cusco | central | 5 | public | 0.00 | 0.00 |
| Universidad Nacional de San Cristóbal de Huamanga | Ayacucho | central | 5 | public | 0.00 | 0.00 |
| Universidad Nacional de San Martín | San Martin | central | 5 | public | 0.00 | 0.00 |
| Universidad Nacional de Trujillo | La Libertad | central | 5 | public | 0.00 | 0.00 |
| Universidad Nacional de Trujillo | La Libertad | filial | 5 | public | 12.50 | 0.00 |
| Universidad Nacional de Trujillo | La Libertad | filial | 5 | public | 12.50 | 0.00 |
| Universidad Nacional de Tumbes | Tumbes | central | 5 | public | 0.00 | 0.00 |
| Universidad Nacional de Ucayali | Ucayali | central | 5 | public | 0.00 | 0.00 |
| Universidad Nacional del Altiplano | Puno | central | 5 | public | 0.00 | 0.00 |
| Universidad Nacional del Callao | Callao | central | 5 | public | 13.75 | 0.00 |
| Universidad Nacional del Centro del Perú | Junín | central | 5 | public | 2.50 | 0.00 |
| Universidad Nacional del Santa | Ancash | central | 5 | public | 22.25 | 0.00 |
| Universidad Nacional Federico Villarreal | Lima | central | 5 | public | 0.00 | 0.00 |
| Universidad Nacional Hermilio Valdizán | Huánuco | central | 5 | public | 15.00 | 25.00 |
| Universidad Nacional Jorge Basadre Grohmann | Tacna | central | 5 | public | 0.00 | 0.00 |
| Universidad Nacional José Faustino Sánchez Carrión | Lima | central | 5 | public | 0.00 | 0.00 |
| Universidad Nacional Mayor de San Marcos | Lima | central | 5 | public | 0.00 | 0.00 |
| Universidad Nacional Santiago Antúnez de Mayolo | Ancash | central | 5 | public | 0.00 | 0.00 |
| Universidad Nacional Toribio Rodríguez de Mendoza de Amazonas | Amazonas | central | 5 | public | 15.00 | 0.00 |
| Universidad Peruana Cayetano Heredia | Lima | central | 5 | private | 1,176.25 | 1,603.75 |
| Universidad Peruana Los Andes | Junín | central | 5 | private | 618.25 | 0.00 |
| Universidad Peruana Unión | Lima | central | 5 | private | 484.75 | 1,723.75 |
| Universidad Privada Antenor Orrego | La Libertad | central | 5 | private | 662.50 | 0.00 |
| Universidad Privada de Huancayo Franklin Roosevelt | Junín | central | 5 | private | 610.00 | 0.00 |
| Universidad Privada del Norte | Lima | filial | 5 | private | 1,022.50 | 0.00 |
| Universidad Privada del Norte | Lima | filial | 5 | private | 1,008.75 | 0.00 |
| Universidad Privada del Norte | Lima | filial | 5 | private | 926.25 | 0.00 |
| Universidad Privada Norbert Wiener | Lima | central | 5 | private | 1,025.00 | 0.00 |
| Universidad Privada San Juan Bautista | Ica | filial | 5 | private | 665.00 | 790.00 |
| Universidad Privada San Juan Bautista | Ica | filial | 5 | private | 665.00 | 790.00 |
| Universidad Privada San Juan Bautista | Lima | central | 5 | private | 882.50 | 1,020.00 |
| Universidad San Martin de Porres | Lima | central | 5 | private | 1,124.75 | 1,510.65 |
| Universidad Señor de Sipán | Lambayeque | central | 5 | private | 662.50 | 0.00 |
| Universidad Tecnológica de los Andes | Apurimac | central | 5 | private | 725.00 | 0.00 |
| Universidad Tecnológica de los Andes | Apurimac | filial | 5 | private | 725.00 | 0.00 |
| Universidad Tecnológica de los Andes | Cusco | filial | 5 | private | 725.00 | 0.00 |
| Universidad Tecnológica del Perú | Lima | central | 5 | private | 1,116.25 | 0.00 |

| **PSYCHOLOGY** | | | | | | |
| --- | --- | --- | --- | --- | --- | --- |
| University | Location | Campus | Duration/ years (y), months(m) | Management type | Minimum Cost by semester (USD) | Maximum Cost by semester (USD) |
| Pontificia Universidad Católica del Perú | Lima | central | 5 | private | 1,415.50 | 5,375.75 |
| Universidad Andina del Cusco | Cusco | central | 5 | private | 1,115.00 | 0.00 |
| Universidad Autónoma de Ica | Ica | central | 5 | private | 557.50 | 782.50 |
| Universidad Autónoma de Ica | Ica | filial | 5 | private | 557.50 | 782.50 |
| Universidad Autónoma del Perú | Lima | central | 5y, 5m | private | 900.00 | 0.00 |
| Universidad Católica de Santa María | Arequipa | central | 5 | private | 1,038.00 | 519.00 |
| Universidad Católica de Trujillo Benedicto XVI | La Libertad | central | 6 | private | 470.00 | 0.00 |
| Universidad Católica San Pablo | Arequipa | central | 5 | private | 1,056.25 | 2,056.25 |
| Universidad Católica Santo Toribio de Mogrovejo | Lambayeque | central | 5 | private | 953.75 | 1,402.25 |
| Universidad Católica Sedes Sapientiae | Lima | filial | 5 | private | 950.00 | 850.00 |
| Universidad Católica Sedes Sapientiae | Junín | filial | 5 | private | 497.25 | 554.25 |
| Universidad Católica Sedes Sapientiae | Lima | central | 5 | private | 1,022.50 | 912.50 |
| Universidad Católica Sedes Sapientiae | San Martin | filial | 5 | private | 522.25 | 579.25 |
| Universidad César Vallejo | Ancash | filial | 5 | private | 587.50 | 650.00 |
| Universidad César Vallejo | Ancash | filial | 5y, 6m | private | 587.50 | 650.00 |
| Universidad César Vallejo | Callao | filial | 5y, 6m | private | 650.00 | 775.00 |
| Universidad César Vallejo | La Libertad | central | 5y, 6m | private | 587.50 | 837.50 |
| Universidad César Vallejo | Lambayeque | filial | 5y, 6m | private | 562.50 | 687.50 |
| Universidad César Vallejo | Lima | filial | 5y, 6m | private | 650.00 | 775.00 |
| Universidad César Vallejo | Lima | filial | 5y, 6m | private | 650.00 | 775.00 |
| Universidad César Vallejo | Lima | filial | 5y, 6m | private | 712.50 | 837.50 |
| Universidad César Vallejo | Piura | filial | 5y, 6m | private | 587.50 | 712.50 |
| Universidad César Vallejo | San Martin | filial | 5y, 6m | private | 712.50 | 0.00 |
| Universidad César Vallejo | San Martin | filial | 5y, 6m | private | 587.50 | 687.50 |
| Universidad Científica del Sur | Lima | central | 5 | private | 4,107.50 | 5,827.50 |
| Universidad Científica del Sur | Lima | filial | 5 | private | 4,107.50 | 5,827.50 |
| Universidad Científica del Sur | Lima | filial | 5 | private | 4,107.50 | 5,827.50 |
| Universidad Continental | Arequipa | filial | 5 | private | 810.00 | 1,006.88 |
| Universidad Continental | Junín | filial | 5 | private | 797.50 | 994.38 |
| Universidad Continental | Cusco | filial | 5 | private | 797.50 | 994.38 |
| Universidad Continental | Lima | filial | 5 | private | 1,046.88 | 1,243.75 |
| Universidad de Ciencias y Humanidades | Lima | central | 5 | private | 932.50 | 0.00 |
| Universidad de Huánuco | Huánuco | central | 5y, 6m | private | 450.45 | 0.00 |
| Universidad de Lima | Lima | central | 6 | private | 2,107.50 | 4,482.50 |
| Universidad de Piura | Lima | filial | 6 | private | 2,133.75 | 0.00 |
| Universidad Femenina del Sagrado Corazón | Lima | central | 6 | private | 947.50 | 3,122.50 |
| Universidad Marcelino Champagnat | Lima | central | 6 | private | 1,216.25 | 1,327.75 |
| Universidad María Auxiliadora | Lima | central | 5 | private | 662.50 | 0.00 |
| Universidad Nacional de Piura | Piura | central | 5 | public | 12.50 | 0.00 |
| Universidad Nacional de San Agustín | Arequipa | central | 5 | public | 0.00 | 0.00 |
| Universidad Nacional de San Antonio Abad del Cusco | Cusco | central | 5 | public | 0.00 | 0.00 |
| Universidad Nacional de Tumbes | Tumbes | central | 5 | public | 0.00 | 0.00 |
| Universidad Nacional Federico Villarreal | Lima | central | 6 | public | 10.00 | 0.00 |
| Universidad Nacional Hermilio Valdizán | Huánuco | central | 5 | public | 15.00 | 25.00 |
| Universidad Nacional Mayor de San Marcos | Lima | central | 6 | public | 0.00 | 0.00 |
| Universidad Nacional Toribio Rodríguez de Mendoza de Amazonas | Amazonas | central | 5 | public | 15.00 | 0.00 |
| Universidad Peruana Cayetano Heredia | Lima | central | 5 | private | 2,756.25 | 3,616.25 |
| Universidad Peruana de Ciencias Aplicadas | Lima | central | 5 | private | 2,420.00 | 4,916.25 |
| Universidad Peruana Los Andes | Junín | central | 5 | private | 694.50 | 0.00 |
| Universidad Peruana Unión | Lima | central | 5 | private | 455.25 | 1,576.25 |
| Universidad Privada Antenor Orrego | La Libertad | central | 5 | private | 762.50 | 0.00 |
| Universidad Privada Antenor Orrego | Piura | filial | 5 | private | 762.50 | 0.00 |
| Universidad Privada de Tacna | Tacna | central | 5 | private | 883.75 | 0.00 |
| Universidad Privada del Norte | Cajamarca | filial | 5 | private | 622.50 | 0.00 |
| Universidad Privada del Norte | La Libertad | central | 5 | private | 798.75 | 0.00 |
| Universidad Privada del Norte | Lima | central | 5 | private | 1,022.50 | 0.00 |
| Universidad Privada del Norte | Lima | filial | 5 | private | 1,022.50 | 0.00 |
| Universidad Privada del Norte | Lima | filial | 5 | private | 1,008.75 | 0.00 |
| Universidad Privada del Norte | Lima | filial | 5 | private | 938.75 | 0.00 |
| Universidad Privada Norbert Wiener | Lima | central | 5 | private | 956.25 | 0.00 |
| Universidad Privada San Juan Bautista | Lima | central | 5 | private | 882.50 | 1,020.00 |
| Universidad Privada San Juan Bautista | Lima | central | 5 | private | 882.50 | 1,020.00 |
| Universidad Privada San Juan Bautista | Ica | filial | 5 | private | 638.75 | 766.25 |
| Universidad Privada San Juan Bautista | Ica | filial | 5 | private | 665.00 | 790.00 |
| Universidad Ricardo Palma | Lima | central | 5 | private | 1,887.50 | 0.00 |
| Universidad San Ignacio de Loyola | Lima | central | 5 | private | 2,155.00 | 3,648.75 |
| Universidad San Martin de Porres | Arequipa | filial | 5 | private | 923.00 | 0.00 |
| Universidad San Martin de Porres | Lambayeque | filial | 5 | private | 818.90 | 978.75 |
| Universidad San Martin de Porres | Lima | central | 5 | private | 1,185.40 | 2,061.90 |
| Universidad Señor de Sipán | Lambayeque | central | 5y, 6m | private | 662.50 | 0.00 |
| Universidad Tecnológica del Perú | Ancash | filial | 5 | private | 640.79 | 0.00 |
| Universidad Tecnológica del Perú | Arequipa | filial | 5 | private | 776.25 | 0.00 |
| Universidad Tecnológica del Perú | Ica | filial | 5 | private | 721.31 | 0.00 |
| Universidad Tecnológica del Perú | Junín | filial | 5 | private | 793.69 | 0.00 |
| Universidad Tecnológica del Perú | Lambayeque | filial | 5 | private | 663.75 | 0.00 |
| Universidad Tecnológica del Perú | Lima | central | 5 | private | 951.50 | 0.00 |
| Universidad Tecnológica del Perú | Lima | filial | 5 | private | 951.50 | 0.00 |
| Universidad Tecnológica del Perú | Lima | filial | 5 | private | 951.50 | 0.00 |
| Universidad Tecnológica del Perú | Lima | filial | 5 | private | 1,166.25 | 0.00 |
| Universidad Tecnológica del Perú | Lima | filial | 5 | private | 951.50 | 0.00 |
| Universidad Tecnológica del Perú | Piura | filial | 5 | private | 732.90 | 0.00 |

| **SOCIAL WORK** | | | | | |
| --- | --- | --- | --- | --- | --- |
| University | Location | Campus | Duration/ years (y), months(m) | Management type | Minimum Cost by semester (USD) |
| Universidad Católica de Santa María | Arequipa | central | 5 | private | 499.00 |
| Universidad Nacional de San Agustín | Arequipa | central | 5 | public | 0.00 |
| Universidad Nacional de San Cristóbal de Huamanga | Ayacucho | central | 5 | public | 0.00 |
| Universidad Nacional de Trujillo | La Libertad | central | 5 | public | 12.50 |
| Universidad Nacional de Trujillo | La Libertad | central | 5 | public | 12.50 |
| Universidad Nacional del Altiplano | Puno | central | 5 | public | 0.00 |
| Universidad Nacional del Centro del Perú | Junín | central | 5 | public | 2.50 |
| Universidad Nacional Federico Villarreal | Lima | central | 5 | public | 0.00 |
| Universidad Nacional José Faustino Sánchez Carrión | Lima | central | 5 | public | 0.00 |
| Universidad Nacional Mayor de San Marcos | Lima | central | 5 | public | 0.00 |
| Universidad Señor de Sipán | Lambayeque | central | 10 | private | 662.50 |

| **SPEECH THERAPY** | | | | | | |
| --- | --- | --- | --- | --- | --- | --- |
| University | Location | Campus | Duration/ years (y), months(m) | Management | Minimum Cost by semester (USD) | Maximum Cost by semester (USD) |
| Universidad Nacional Federico Villarreal | Lima | central | 5 | public | 0.00 | 0.00 |
| Universidad Peruana Cayetano Heredia | Lima | central | 10 | private | 1,326.25 | 1,678.75 |

| **OCUPATIONAL THERAPY** | | | | | | |
| --- | --- | --- | --- | --- | --- | --- |
| University | Location | Campus | Duration/ years (y), months(m) | Management | Minimum Cost by semester (USD) | Maximum Cost by semester (USD) |
| Universidad Nacional Mayor de San Marcos | Lima | central | 5 | public | 0.00 | 0.00 |
| Universidad Peruana Cayetano Heredia | Lima | central | 10 | private | 1,326.25 | 1,678.75 |
