# Supplementary material for: Characteristics of undergraduate and second speciality mental health programmes in Peru: a cross-sectional study
Source: Hum Resour Health. 2023 Mar 1;21:16. doi: 10.1186/s12960-023-00805-9 (PMC9979569; doi:10.1186/s12960-023-00805-9)
Supplement: Supplementary file 2 — Additional file 2. Characteristics of second speciality programmes in dollars. [file 12960_2023_805_MOESM2_ESM.docx]

Additional file 2. Characteristics of second speciality and subspecialty programmes in dollars.

| **MEDICINE** | | | | | | |
| --- | --- | --- | --- | --- | --- | --- |
| Program | University | Location | Duration/ years (y), months(m) | Management | Cost for study (USD) | Total cost (USD) |
| Second specialty in Psychiatry | Universidad Católica Santa María | Arequipa | 3 | private | 11,473.00 | 11,823.50 |
| Second specialty in Community Family Medicine | Universidad Católica Santa María | Arequipa | 3 | private | 11,473.00 | 11,986.00 |
| Second specialty in Psychiatry | Universidad de San Martín de Porres | Lima | 3 | private | 8,322.30 | 8,610.80 |
| Second specialty in Community Family Medicine | Universidad de San Martín de Porres | Lima | 3 | private | 8,322.30 | 8,797.80 |
| Second specialty in Psychiatry | Universidad Nacional de Trujillo | Trujillo | 3 | public | 3,075.00 | 3,390.50 |
| Second specialty in Community Family Medicine | Universidad Nacional de Trujillo | Trujillo | 3 | public | 3,075.00 | 3,528.00 |
| Second specialty in Psychiatry | Universidad Nacional Federico Villarreal | Lima | 3 | public | 2,895.00 | 3,233.00 |
| Second specialty in Community Family Medicine | Universidad Nacional Federico Villarreal | Lima | 3 | public | 2,895.00 | 3,395.50 |
| Second specialty in Psychiatry | Universidad Nacional Mayor de San Marcos | Lima | 3 | public | 4,875.00 | 5,175.50 |
| Second specialty in Community Family Medicine | Universidad Nacional Mayor de San Marcos | Lima | 3 | public | 4,875.00 | 5,338.00 |
| Second specialty in Psychiatry | Universidad Peruana Cayetano Heredia | Lima | 3 | private | 9,090.00 | 9,408.00 |
| Second specialty in Community Family Medicine | Universidad Peruana Cayetano Heredia | Lima | 3 | private | 8,820.00 | 9570.50 |
| Second specialty in Psychiatry | Universidad Privada Antenor Orrego | Trujillo | 3 | private | 7,950.00 | 8,303.00 |
| Second specialty in Community Family Medicine | Universidad Privada Antenor Orrego | Trujillo | 3 | private | 7,950.00 | 8,465.50 |
| Second specialty in Psychiatry | Universidad Privada San Juan Bautista | Lima | 3 | private | 5,350.00 | 8,413.00 |
| Second specialty in Community Family Medicine | Universidad Privada San Juan Bautista | Lima | 3 | private | 5,350.00 | 8,575.50 |
| Second specialty in Psychiatry | Universidad Ricardo Palma | Lima | 3 | private | 7,425.00 | 7,713.00 |
| Second specialty in Community Family Medicine | Universidad Ricardo Palma | Lima | 3 | private | 7,425.00 | 7,875.50 |
| Second specialty in Community Family Medicine | Universidad Nacional del Altiplano | Puno | 3 | public | 4,050.00 | 4,425.50 |
| Second specialty in Community Family Medicine | Universidad Científica del Sur | Lima | 3 | private | 8,730.00 | 9,255.50 |
| Second specialty in Community Family Medicine | Universidad Peruana de los Andes | Junín | 3 | private | 9,900.00 | 10,450.50 |
| Second specialty in Community Family Medicine | Universidad César Vallejo | Trujillo | 3 | private | 6,862.50 | 7,355.50 |
| Second specialty in Community Family Medicine | Universidad Nacional de Piura | Piura | 3 | public | 3,446.63 | 3,822.13 |
| Second specialty in Community Family Medicine | Universidad Nacional del Centro del Perú | Junín | 3 | public | 11,250.00 | 11,730.50 |
| Second specialty in Community Family Medicine | Universidad peruana de Ciencias Aplicadas | Lima | 3 | private | 9,090.00 | 9,570.50 |
| Second specialty in Psychiatry* | Universidad Nacional de San Agustín | Arequipa | 3 | public | 3,975.00 | 0.00 |
| Second specialty in Community Family Medicine* | Universidad Nacional de San Agustín | Arequipa | 3 | public | 3,975.00 | 0.00 |
| Second specialty in Psychiatry* | Universidad Privada de Tacna | Tacna | 3 | private | 9,450.00 | 0.00 |
| Second specialty in Community Family Medicine* | Universidad Privada de Tacna | Tacna | 3 | private | 9,450.00 | 0.00 |
| Second specialty in Community Family Medicine* | Universidad Nacional de San Antonio Abad del Cusco | Cusco | 3 | public | 1,068.00 | 0.00 |
| Second specialty in Community Family Medicine* | Universidad Nacional de la Amazonía Peruana | Loreto | 3 | public | 0.00 | 0.00 |

| **NURSING** | | | | | | |
| --- | --- | --- | --- | --- | --- | --- |
| Program | University | Location | Duration/ years (y), months(m) | Management | Cost for study (USD) | Total cost (USD) |
| Second interdisciplinary specialty with mention in: health, mental health and psychiatry. | Universidad Nacional de Ucayali | Ucayali | 1 | public | 1,104.53 | 1,129.37 |
| Second professional specialty in mental health nursing | Universidad Nacional del Callao | Callao | 8m | public | 700.00 | 720.00 |
| Second professional specialty in public and community health | Universidad Nacional del Callao | Callao | 8m | public | 700.00 | 720.00 |
| Mental Health and Psychiatric Nursing | Universidad Nacional Federico Villarreal | Lima | 1y, 4m | public | 1,500.00 | 1,575.00 |
| Second Nursing Specialty in Public and Community Health | Universidad Nacional Federico Villarreal | Lima | 1y, 4m | public | 1,500.00 | 1,575.00 |
| Second Nursing Specialty in Mental Health and Psychiatry | Universidad Nacional Mayor de San Marcos | Lima | 1y, 4m | public | 2,000.00 | 2,140.00 |
| Second Public Health Nursing Specialty | Universidad Nacional Mayor de San Marcos | Lima | 1y, 4m | public | 2,000.00 | 2,140.00 |
| Second Specialty of Nursing in Mental Health and Psychiatry | Universidad Peruana Cayetano Heredia | Lima | 1y, 4m | private | 3,375.00 | 3,487.50 |
| Second Specialty of Nursing in Mental Health and Psychiatry | Universidad Privada Norbert Wiener | Lima | 1 | private | 1,725.00 | 1,800.00 |
| Second Specialty of Nursing in Public Health and Health Services Management | Universidad Nacional de San Antonio Abad del Cusco | Cusco | 1 | public | 1,000.00 | 1,078.25 |
| Second Specialty in Public and Community Health Nursing | Universidad Nacional Jorge Basadre Grohmann | Tacna | 8m | public | 785.00 | 810.00 |
| Second Specialty in Family and Community Health Nursing | Universidad de San Martín de Porres | Lima | 10m | private | 1,158.75 | 1,183.75 |
| Second Public Health Nursing Specialty | Universidad de San Martín de Porres | Lima | 10m | private | 1,158.75 | 1,183.75 |
| Second specialty in mental health nursing and psychiatry | Universidad Nacional de la Amazonía Peruana | Iquitos | 1y, 6m | public | 0.00 | 0.00 |
| Second Professional Specialty in Public and Community Health Nursing | Universidad Nacional de la Amazonía Peruana | Iquitos | NA | public | 0.00 | 0.00 |
| Second nursing specialty in Mint Health | Universidad Nacional de San Agustín | Arequipa | 1 | public | 0.00 | 0.00 |

| **PSYCHOLOGY** | | | | | | |
| --- | --- | --- | --- | --- | --- | --- |
| Program | University | Location | Duration/ years (y), months(m) | Management | Cost for study (USD) | Total cost (USD) |
| Systemic Family Psychotherapy | Universidad Nacional Federico Villarreal | Lima | 1y, 2m | public | 2,600.00 | 2,637.50 |
| Cognitive Behavioural Therapy | Universidad Nacional Federico Villarreal | Lima | 1y, 2m | public | 2,600.00 | 2,637.50 |
| Second speciality in Family Therapy and Systematic Intervention with a focus on violence and addictions | Universidad Católica de Trujillo Benedicto XVI | Trujillo | 1 | private | 850.00 | 875.00 |
| Second speciality in Family Therapy and Systematic Intervention with a focus on couples and sexuality | Universidad Católica de Trujillo Benedicto XVI | Trujillo | 1 | private | 850.00 | 875.00 |

| **MULTIDISCIPLINARY PROGRAMMES** | | | | | | |
| --- | --- | --- | --- | --- | --- | --- |
| Program | University | Location | Duration/ years (y), months(m) | Management | Cost for study (USD) | Total cost (USD) |
| Second Professional Specialisation in Assessment and Intervention Strategies for Children's Language Difficulties | Universidad Nacional Mayor de San Marcos | Lima | 2 | public | 1,712.00 | 1,737.00 |
| Second speciality in special education: hearing and language | Universidad Femenina del Sagrado Corazón | Lima | NA | private | 815.00 | 867.50 |
| Second speciality in speech therapy and integrated speech therapy | Universidad Privada de Tacna | Tacna | 1 | private | 0.00 | 0.00 |
| Second speciality in education speciality: audition and language | Universidad Católica de Trujillo Benedicto XVI | Trujillo | 1 | private | 800.00 | 825.00 |

| **MEDICINE** | | | | | | |
| --- | --- | --- | --- | --- | --- | --- |
| Program | University | Location | Duration/ years (y), months(m) | Management | Cost for study (USD) | Total cost (USD) |
| Subspecialty in Addiction Psychiatry | Universidad de San Martín de Porres | Lima | 2 | private | 5,548.20 | 5,861.20 |
| Subspecialty in Child and Adolescent Psychiatry | Universidad de San Martín de Porres | Lima | 2 | private | 5,548.20 | 5,861.20 |
| Subspecialty in Child and Adolescent Psychiatry | Universidad Nacional Federico Villarreal | Lima | 2 | public | 1,930.00 | 2,268.00 |
| Subspecialty in Child and Adolescent Psychiatry | Universidad Nacional Mayor de San Marcos | Lima | 2 | public | 3,250.00 | 3,550.50 |
| Subspecialty in Addiction Psychiatry | Universidad Nacional Mayor de San Marcos | Lima | 2 | public | 3,250.00 | 3,550.50 |
| Subspecialty in Child and Adolescent Psychiatry | Universidad Peruana Cayetano Heredia | Lima | 2 | private | 8,820.00 | 9,138.00 |
| Subspecialty in Child and Adolescent Psychiatry | Universidad Ricardo Palma | Lima | 2 | private | 4,950.00 | 5,238.00 |
